# Prognostic value of neutrophil to lymphocyte ratio for patients with bladder cancer undergoing radical cystectomy: a systematic review and meta-analysis

**DOI:** 10.3389/fonc.2024.1463173

**Published:** 2024-10-24

**Authors:** Zhan Chen, Yao Zhang, Telei Chen

**Affiliations:** ^1^ Department of Urology, Cixilntegrated Traditional Chinese and Western Medicine Medical, Ningbo, Zhejiang, China; ^2^ Department of Urology, Ningbo Yinzhou No.2 Hospital, Ningbo, Zhejiang, China

**Keywords:** neutrophil, lymphocyte, NLR, bladder cancer, radical cystectomy, meta-analysis

## Abstract

**Objectives:**

This study evaluated the prognostic value of the neutrophil-to-lymphocyte ratio (NLR) for survival outcomes in bladder cancer patients treated with radical cystectomy.

**Methods:**

Studies assessing NLR’s prognostic significance for bladder cancer after radical cystectomy were identified from PubMed, Embase, Web of Science, and Cochrane databases until April 2024. Survival outcomes analyzed included overall survival (OS), disease-free survival (DFS), relapse-free survival (RFS), cancer-specific survival (CSS), and progression-free survival (PFS).

**Results:**

The meta-analysis comprised 15 cohort studies with 8,448 patients. Multivariate analysis showed significantly shorter OS, CSS, DFS, and RFS in the high NLR group compared to the low NLR group. However, no significant difference in PFS was observed between the groups.

**Conclusions:**

NLR serves as an independent prognostic indicator for bladder cancer patients undergoing radical cystectomy, with elevated NLR associated with poorer survival. Further large-scale, prospective studies are warranted to validate the relationship between NLR and prognosis in bladder cancer.

**Systematic review registration:**

https://www.crd.york.ac.uk/PROSPERO/, identifier CRD42024549573.

## 1 Introduction

Bladder cancer, with urothelial carcinoma as the primary pathological type, ranks among the most prevalent malignancies affecting the urinary system. Up to 70-85% of patients with bladder cancer are initially diagnosed as non-muscle invasive bladder cancer (NMIBC), while 15-30% are diagnosed as or progress to muscle-invasive bladder cancer (MIBC) (1). The initial treatment strategy of patients with NMIBC is the transurethral resection of bladder tumor (TURBT), followed by intravesical therapy of bacillus Calmette–Guerin (BCG) or chemotherapy (according to grade and focality of the tumor). However, current studies have shown that different adjuvant intravesical BCG strains have no significant advantage in reducing the recurrence rate of bladder cancer (1), and 20% to 40% of these tumors may manifest as or progress to muscle-invasive cancer, a highly malignant form prone to early metastasis (2, 3).

In MIBC, neoadjuvant chemotherapy followed by radical cystectomy with bilateral pelvic lymphadenectomy represents the mainstay of treatment, encompasses three main surgical approaches: traditional open radical cystectomy (ORC), laparoscopic radical cystectomy (LRC), and robot-assisted radical cystectomy (RARC) (4, 5). Compared to ORC, LRC is effective in reducing intraoperative blood loss and injury, accelerating postoperative recovery, and significantly decreasing total postoperative complications (4, 5). Although RARC further diminishes intraoperative bleeding and surgical complication rates, it shows no significant advantage over LRC and substantially increases hospitalization costs (6). Currently, LRC is the most prevalent surgical modality for non-metastatic MIBC, offering certain benefits over ORC. However, the prognosis remains poor, with a 5-year postoperative survival rate of only about 50% (7, 8). In addition, for patients with MIBC, the standard neoadjuvant treatment before cystectomy is three or four cycles of cisplatin chemotherapy. Although guidelines recommend four cycles of cisplatin-gemcitabine treatment, three cycles of treatment are often used in clinical practice, and the survival difference between different neoadjuvant treatment regimens seems to be inconclusive (9). Thus, identifying independent predictors of survival risk in these patients, screening those who can benefit more from radical cystectomy, and closely monitoring high-risk patients for individualized treatment holds great clinical significance.

Numerous studies have established that the systemic inflammatory response significantly influences tumor occurrence and progression, serving as an independent factor impacting cancer patients’ prognosis (10, 11). In-depth studies on the direct or indirect interaction between tumor cells and inflammatory factors, such as neutrophils, lymphocytes, monocytes, platelets, and C-reactive protein, have provided increasing evidence that the systemic inflammatory response affects patient prognosis by altering the tumor cell microenvironment (12–14). Several studies have shown that non-steroidal anti-inflammatory drugs can significantly reduce the risk of bladder cancer, stomach cancer, endometrial cancer, and other malignant tumors, while significantly improving patient prognosis (15–17). Neutrophils are essential for neovascularization through a series of enzymatic reactions, which also forms the basis for distant tumor metastasis via blood transport (18, 19). Based on extensive basic research, the human immune process involving lymphocytes has a certain inhibitory effect on tumor occurrence and development (20). Neutrophil to lymphocyte ratio (NLR), an effective indicator of systemic inflammatory response, can reflect body inflammation in clinical practice. Numerous studies have shown that a lower pre-surgery NLR level predicts better prognosis in various malignant tumors, such as gastric cancer, colon cancer, and small cell lung cancer, compared to patients with high NLR levels (21–26).

While numerous studies have reported the predictive value of NLR for prognosis in bladder cancer patients, conclusive evidence regarding its effectiveness in predicting long-term outcomes following radical cystectomy remains elusive due to variations in study duration, cancer types, treatment modalities, detection timing, and other factors (27–41). The present study aimed to investigate the clinical utility of NLR in predicting long-term prognosis after radical cystectomy for bladder cancer patients through a systematic review and meta-analysis, thereby providing the latest and most comprehensive evidence-based medical foundation for developing accurate postoperative prognosis prediction models in this patient population.

## 2 Methods

### 2.1 Literature search

In line with the PRISMA 2020 statement (42), this meta-analysis was conducted and prospectively registered in the PROSPERO database (CRD42024549573). A systematic literature search was conducted in PubMed, Embase, Web of Science, and Cochrane databases until April 2024 to identify studies evaluating the prognostic value of postoperative NLR in predicting survival outcomes for bladder cancer patients after radical cystectomy. The literature search used the following terms: “bladder neoplasms”, “lymphocytes”, and “neutrophil”. The detailed search strategies were: ((((“Neutrophils”[Mesh]) OR (((Neutrophil) OR (Neutrophil Band Cells)) OR (Neutrophil Band Cell))) AND ((“Lymphocytes”[Mesh]) OR (((((Lymphocyte) OR (Lymphoid Cells)) OR (Cell, Lymphoid)) OR (Cells, Lymphoid)) OR (Lymphoid Cell)))) AND (ratio)) AND ((“Urinary Bladder Neoplasms”[Mesh]) OR ((((((Bladder Tumor) OR (Urinary Bladder Neoplasm)) OR (Bladder Neoplasm)) OR (Urinary Bladder Cancer)) OR (Bladder Cancer)) OR (Cancer of Bladder)))). Additionally, the reference lists of included studies were manually screened. Two authors independently retrieved and evaluated eligible articles, resolving any discrepancies through discussion.

### 2.2 Inclusion and exclusion criteria

Eligible studies fulfilled the following criteria: (1) randomized controlled trials, cohort studies, or case-control studies; (2) included bladder cancer patients who underwent radical cystectomy; (3) evaluated the prognostic significance of NLR for survival outcomes; (4) assessed at least one survival outcome measure [overall survival (OS), disease-free survival (DFS), relapse-free survival (RFS), progression-free survival (PFS), or cancer-specific survival (CSS)]; (5) provided adequate data for multivariate analysis of risk ratios (RR), odds ratios (OR), or hazard ratios (HR). Study protocols, unpublished research, non-original articles (letters, comments, abstracts, errata, replies), studies with insufficient data, and review articles were excluded from consideration.

### 2.3 Data abstraction

Two authors independently abstracted data from eligible studies, with any discrepancies resolved by a third author. The extracted information included first author name, year of publication, study duration, country, study design, study population, treatment modality, sample size, follow-up duration, timing of NLR assessment, age, gender, TNM stage, NLR cut-off value, and multivariate analysis (including Multivariate analysis variables) hazard ratios (HRs) for overall survival (OS), disease-free survival (DFS), relapse-free survival (RFS), and cancer-specific survival (CSS). In cases where study data were incomplete, the corresponding authors were contacted to obtain the missing information.

### 2.4 Quality evaluation

The Newcastle-Ottawa Scale (NOS) (43), was used to assess the quality of included cohort studies, with scores of 7-9 considered high quality (44). Studies with NOS scores <7 were excluded from quantitative analysis. Two authors independently evaluated the quality of included studies, resolving discrepancies through discussion.

### 2.5 Statistical analysis

The meta-analysis was performed using Review Manager 5.4.1. Survival data were synthesized using hazard ratios (HRs) and presented with 95% confidence intervals (CIs). Heterogeneity for each outcome was assessed using Cochran’s Q (chi-squared test) and I^2^ inconsistency index (45), with high heterogeneity defined as a P-value <0.1 or I^2^ >50%. The overall HR for each outcome was calculated using a random-effects model. Sensitivity analysis was conducted for results with ≥3 studies to evaluate each study’s impact on the overall HR. For outcomes with ≥10 studies, potential publication bias was assessed by generating funnel plots in Review Manager 5.4.1 and performing Egger’s regression tests (46) using Stata 15.1 (Stata Corp, College Station, Texas, USA). A *P*-value <0.05 indicated statistically significant publication bias.

## 3 Results

### 3.1 Literature retrieval, study characteristics, and baseline

**Figure 1:**
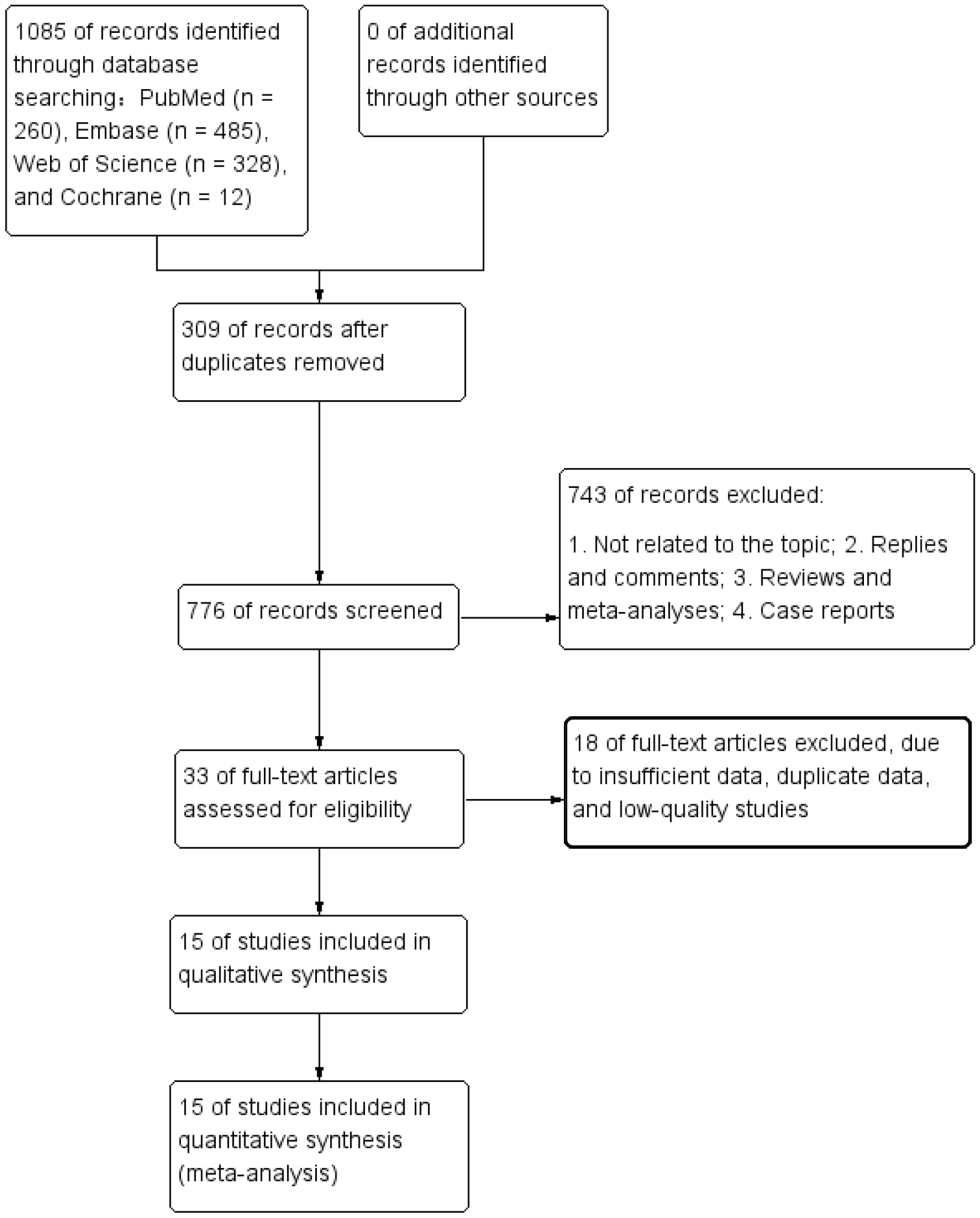
Flowchart of the systematic search and selection process.

The literature retrieval and selection process is depicted in **Figure 1**. A systematic literature search identified 1,085 related studies in PubMed (n = 260), Embase (n = 485), Web of Science (n = 328), and Cochrane (n = 12). After duplicate removal, 776 titles and abstracts were assessed. The meta-analysis included 15 retrospective cohort studies with 8,448 patients (27–41). Characteristics and quality evaluation of eligible cohort studies are presented in **Table 1**.

**Table 1:** Baseline characteristics of include studies and methodological assessment.

Study	Study period	Country	Study design	Population	No. of patients	Median follow-up	Gender		Median/mean age	NLR cut-off	Multivariable analysis	NOS score
							Male	Female				
D'Andrea 2017	1990-2012	Multi-center	Retrospective cohort	Patients undergoing RC for clinically nonmetastatic UCB	4335	NA	3362	836	67	2.7	Gender, age, pathologic stage, positive soft tissue surgical margin, concomitant carcinoma in situ, lymph node involvement, lymphovascular invasion, adjuvant chemotherapy, lymphocyte-to-monocyte ratio	8
Hermanns 2014	1992-2012	Canada	Retrospective cohort	Patients undergoing radical cystectomy for urothelial carcinoma of the bladder	424	58.4 months	325	99	70.1	3	Age, gender, Charlson comorbidity index, haemoglobin, platelets, T-stage, N-Stage, positive surgical margin, year of radical cystectomy, lymphovascular invasion, NAC/primary chemotherapy, adjuvant chemotherapy	7
Herzberg 2022	2010-2020	Israel	Retrospective cohort	Patients with bladder cancer who underwent radical cystectomy	346	44 months	268	78	69	2.54	NA	7
Kang 2016	1999-2012	Korea	Retrospective cohort	Patients with urothelial carcinoma of the bladder undergoing radical cystectomy	385	38 months	NA	NA	NA	2	Age, BMI, pT stage, tumor grade, presence of lymphovascular invasion, presence of variant form, surgical margin status, No. of lymph nodes removed, lymph node status, adjuvant chemotherapy	7
Kawahara 2016	1999-2014	Japan	Retrospective cohort	Patients who underwent radical cystectomy	74	24.2 months	58	16	65	2.38	Age, gender, ECOG-PS, CPR, LDH, neoadjuvant chemotherapy, clinical T stage, clinical lymph node metastasis, surgical margin, adjuvant chemotherapy, pathological T stage, pathological lymph node metastasis	9
Morizawa 2016	2002-2013	Japan	Retrospective cohort	Patients who underwent RC for muscle-invasive bladder cancer	110	37.5 months	86	24	72	2.6	ECOG-PS, hydronephrosis, pathological T stage, NAC, lymph node metastasis, tumor growth pattern	7
Ozcan 2015	1990-2013	Turkey	Retrospective cohort	Patients who underwent radical cystectomy for bladder cancer	286	28 months	256	30	60.7	2.5	Age, gender, lymph node invasion, pathological stage, histologic type, surgical margin, grade, lymphovascular invasion, carcinoma in-situ, preoperative hydronephrosis, leukocyte, neutrophil, lymphocyte	8
Peng 2017	2006-2012	China	Retrospective cohort	Patients with bladder cancer who underwent radical cystectomy	516	37 months	436	80	66	NA	Age, gender, histology type, pathological grade, pT, pN status, smoking history, hypertension, diabetes mellitus, heart disease, cerebrovascular disease, ASA grade, hypoalbuminemia, anemia, postoperative complications	7
Sudoł 2022	2011-2017	Poland	Retrospective cohort	Patients with BC who underwent RC	134	2.2 years	115	19	66	2.7	Age, pathologic tumor stage, lymphovascular invasion, positive radial margin, urinary diversion	8
Tan 2017	2002-2012	Singapore	Retrospective cohort	Patients undergoing radical cystectomy for bladder cancer	84	30.1 months	63	21	67	2.7	Size, No. of tumors, high grade tumor, hydronephrosis, anemia, T3/4 disease, LN involvement	8
Viers 2014	1994-2005	USA	Retrospective cohort	Patients with urothelial carcinoma of the bladder undergoing radical cystectomy	899	10.9 years	723	176	69	2.7	Age at surgery, sex, ECOG performance status, clinical tumor stage, carcinoma in situ, hydronephrosis preoperative BCG treatment, lymphovascular invasion	8
von Deimling 2023	NA	Multi-center	Retrospective cohort	Muscle invasive bladder cancer treated with neoadjuvant chemotherapy and radical cystectomy	404	49 months	298	106	65	NA	Age, gender, smoking history, any secondary variant histology, cisplatin-based NAC regimen, number of NAC cycles, clinical tumor stage, concomitant CIS at TURBT, clinically N + disease, C-index of full model	7
Zattoni 2023	2013-2016	Italy	Retrospective cohort	Patients with urothelial carcinoma of the bladder following radical cystectomy	132	15.9 months	105	27	74	2.7	Age at surgery, sex, ECOG performance status, intravesical therapy	7
Zhang 2020	2007-2018	China	Retrospective cohort	Patients with MIBC who underwent RC	202	30 months	168	34	66.1	2.42	Age, pathological T stage, pathological grade, lymph node	8
Zhang 2021	2005-2017	China	Retrospective cohort	Bladder cancer patients after radical cystectomy	127	NA	110	17	66	3.73	Sex, age, BMI, smoking, cardiopulmonary disease, diabetes mellitus, tumor size, T stage, tumor histological types, grade, albumin, hemoglobin	8

NA, not available.

### 3.2 OS

**Figure 2:**
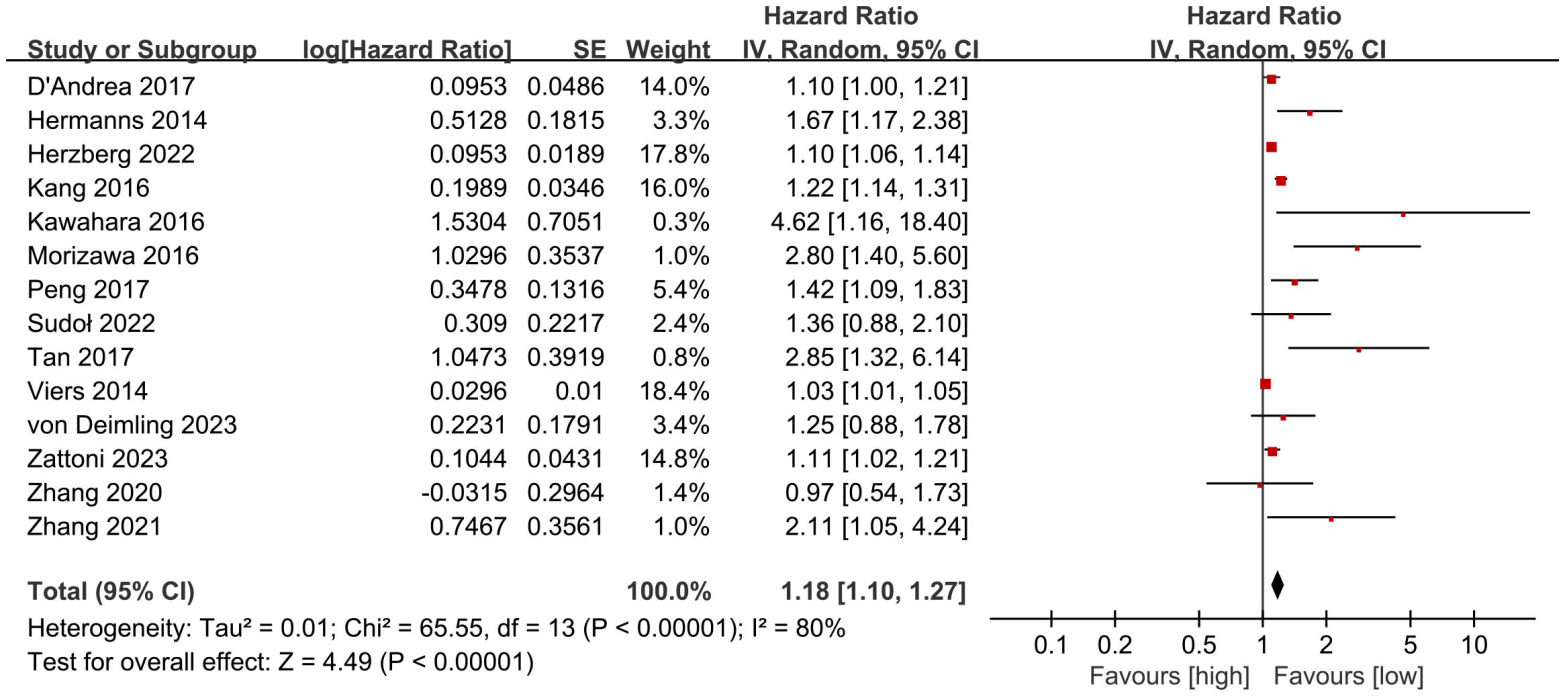
Forest plots of OS.

OS results were synthesized from 14 cohort studies (27–38, 40, 41). Meta-analysis of multivariate data showed significantly shorter OS in the high NLR group compared to the low NLR group (HR: 1.18; 95% CI: 1.10, 1.27; *P <*0.00001). Significant heterogeneity was observed (I^2^ = 80%, *P <*0.00001) (**Figure 2**).

### 3.3 CSS

**Figure 3:**
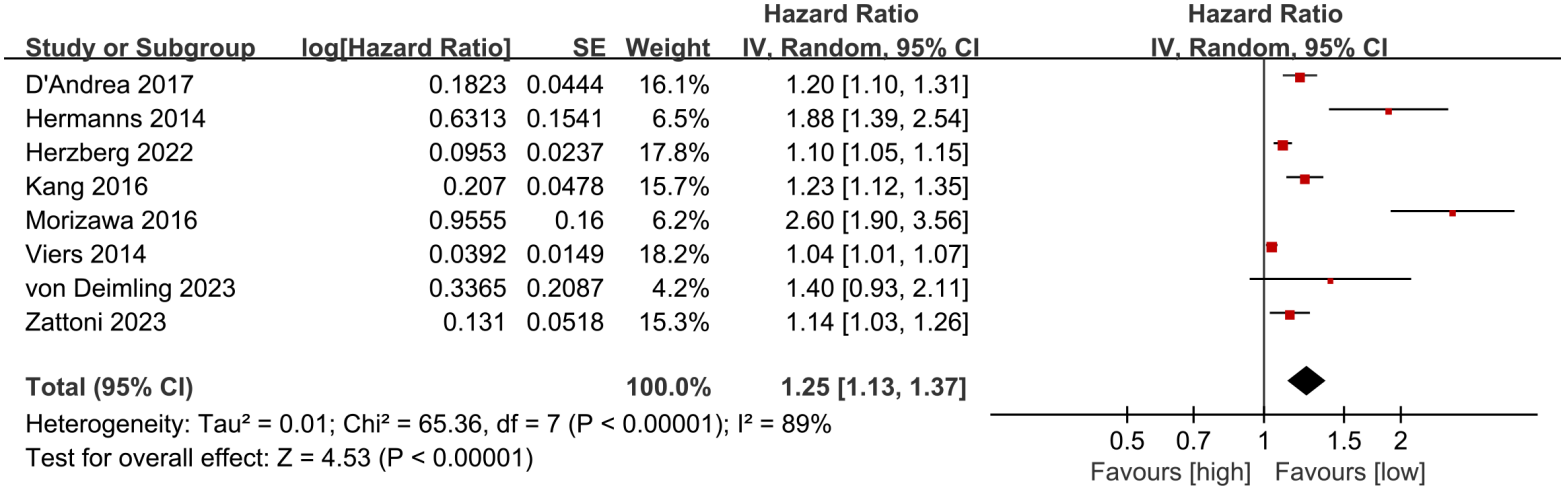
Forest plots of CSS.

CSS results were synthesized from 8 cohort studies (27, 28, 30, 35, 36, 38, 40, 41). Meta-analysis of multivariate data showed significantly shorter CSS in the high NLR group compared to the low NLR group (HR: 1.25; 95% CI: 1.13, 1.37; P <0.00001). Significant heterogeneity was observed (I^2^ = 89%, *P <*0.00001) (**Figure 3**).

### 3.4 RFS

**Figure 4:**
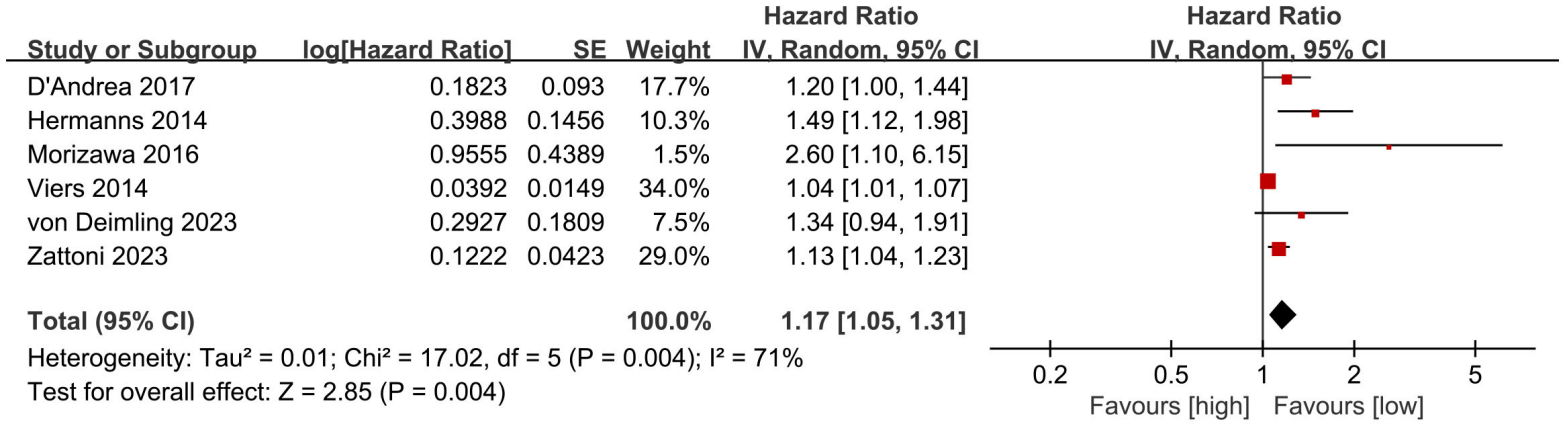
Forest plots of RFS.

RFS results were synthesized from 6 cohort studies (27, 28, 35, 36, 40, 41). Meta-analysis of multivariate data showed significantly shorter RFS in the high NLR group compared to the low NLR group (HR: 1.17; 95% CI: 1.05, 1.31; *P* = 0.004). Significant heterogeneity was observed (I^2^ = 71%, *P* = 0.004) (**Figure 4**).

### 3.5 DFS

**Figure 5:**
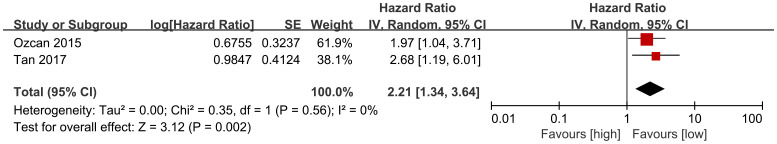
Forest plots of DFS.

DFS results were synthesized from 2 cohort studies (33, 39). Meta-analysis of multivariate data showed significantly shorter DFS in the high NLR group compared to the low NLR group (HR: 2.21; 95% CI: 1.34, 3.64; P = 0.002). No significant heterogeneity was observed (I^2^ = 0%, *P* = 0.56) (**Figure 5**).

### 3.6 PFS

PFS results were synthesized from 2 cohort studies (31, 33). Meta-analysis of multivariate data showed similar PFS between the two groups (HR: 1.27; 95% CI: 0.83, 1.95; *P* = 0.27). Significant heterogeneity was observed (I^2^ = 64%, *P* = 0.10) (**Supplementary Figure S1**).

### 3.7 Publication bias and sensitivity analysis

Potential publication bias for OS was evaluated using funnel plots and Egger’s regression tests. Significant publication bias for OS was detected visually (funnel plots) and statistically (Egger’s test *P* = 0.0001) (**Supplementary Figure S2**). Sensitivity analysis was conducted for OS, CSS, and RFS results to evaluate each cohort study’s impact on the overall HR by sequential exclusion. Sensitivity analysis revealed stable overall HRs after excluding each cohort study for OS (**Supplementary Figure S3A**), CSS (**Supplementary Figure S3B**), and RFS (**Supplementary Figure S3C**).

## 4 Discussion

For locally resectable MIBC without distant metastasis, radical cystectomy and pelvic lymph node dissection can improve survival and avoid local recurrence and distant metastasis (47). Currently, patient prognosis is mainly evaluated based on bladder tumor stage, grade, and local lymph node metastasis, with host systemic inflammatory response rarely considered (48–50). Studies have revealed that bladder tumor occurrence relates to long-term chronic inflammation, and long-term NSAID use can reduce bladder cancer incidence (17). In recent years, systemic inflammatory response and tumor occurrence/development have become a research hotspot, with evidence showing systemic inflammatory response’s involvement in cancer progression, promoting tumor occurrence/development at various stages, and enhancing tumor cell proliferation, invasion, metastasis, and anti-apoptosis (51, 52). Using systemic inflammatory response indicators to predict tumor recurrence and progression after surgery is clinically significant (64).

This meta-analysis evaluated the prognostic value of NLR for survival outcomes in bladder cancer patients undergoing radical cystectomy. Results showed significantly shorter OS, CSS, RFS, and DFS in the high NLR group compared to the low NLR group, suggesting NLR’s predictive value for prognosis in bladder cancer patients undergoing radical cystectomy, warranting attention in clinical treatment. Our findings support most previous research (53–57). Ofner et al. conducted a meta-analysis of the NLR-RFS relationship using studies before 2022 (53), including seven articles. Results showed a statistically significant association between elevated NLR and increased recurrence risk (53). This finding aligns with our conclusions, and we additionally found significant NLR associations with OS, CSS, and DFS, providing the most comprehensive evidence for using NLR to predict long-term prognosis after radical bladder cancer surgery.

Studies have shown tumor-associated neutrophils (TAN) significantly influence tumor biology. Based on activation pathways, TAN categorize into N1 (anti-tumor) and N2 (pro-tumor) types. In tumor tissues, N2 TAN secrete angiogenic factors, chemokines, cytokines, and reactive oxygen species, promoting tumor development (58). Bladder cancer cells secrete granulocyte colony-stimulating factors, leading to increased neutrophil production. These neutrophils facilitate the formation of new blood vessels by releasing elastase, breaking down histones, and degrading the extracellular matrix, thereby promoting tumor cell proliferation and metastatic spread (59). Thus, increased neutrophils in tumor patients closely associate with tumor progression. An effective anti-tumor immune response requires presence, activation, and co-stimulation of immune system lymphoid components, including CD8+T cells, B cells, and intrinsic lymphocytes (60). Recent immune studies have demonstrated lymphocytes, the immune core, participate in cellular and humoral immunity *in vivo* (61, 62). Decreased lymphocyte number in tumor tissue reduces local immune function, creating an immune-impaired environment favoring tumor growth. Additionally, tumor microenvironment components inhibit lymphocyte differentiation and maturation, leading to lymphocyte function loss and depletion (63).

With tumor invasion and metastasis, the body’s immune function is inhibited, and lymphocyte proliferation in early stages is inhibited in later stages, resulting in reduced lymphocyte differentiation, maturation, and number (63–65). With malignant tumor progression, cancer cell infiltration manifests as a stronger inflammatory response, and neutrophils increase accordingly (63, 65). With later tumor stage and lymph node metastasis, NLR is higher and patient prognosis worse. Celik et al. (66) showed in bladder cancer patients with largest tumor diameter >3cm, NLR level differed by stage, and preoperative NLR level helped judge tumor stage. Zhang et al. (32) retrospectively analyzed 202 MIBC patients undergoing radical surgery, finding late pathological stage and positive lymph node status as risk factors for PFS and OS, while high NLR was a risk factor for OS.

This study revealed the significance of NLR, a commonly used hematological index, for prognosis in bladder cancer patients undergoing radical cystectomy, with certain limitations. Firstly, no unified standard exists for selecting and calculating the optimal NLR cut-off value. ROC curve and median methods are commonly used. Different NLR cut-off calculation methods across included studies, with varying case numbers and cut-off values, led to heterogeneity in results. Secondly, while all subjects were bladder cancer patients undergoing radical cystectomy, included studies differed in patient characteristics, clinical stages, pathological types, tumor invasion, surgical methods, and adjuvant therapy. Additionally, included studies were retrospective with small sample sizes, mostly single-center data from Europe and Asia, inevitably leading to selective bias. Despite limitations, this meta-analysis is the most recent and comprehensive evidence-based study reporting NLR’s prognostic value for survival outcomes in bladder cancer patients undergoing radical cystectomy. Results support focusing on NLR level changes in bladder cancer clinical treatment, especially post-surgery, and establishing a predictive model based on factors including NLR to improve post-surgery long-term survival.

## 5 Conclusion

Multivariate meta-analysis demonstrated NLR as an independent prognostic factor in bladder cancer patients undergoing radical cystectomy, with high NLR associated with poor prognosis. Given retrospective study limitations, potential selection bias, and heterogeneity, large-scale, multicenter, prospective clinical studies are needed to further validate the NLR-bladder cancer prognosis relationship.

## Data Availability

The original contributions presented in the study are included in the article/**Supplementary Material**. Further inquiries can be directed to the corresponding author.
