# Supplementary material for: Prognostic value of neutrophil to lymphocyte ratio for patients with bladder cancer undergoing radical cystectomy: a systematic review and meta-analysis
Source: Front Oncol. 2024 Oct 24;14:1463173. doi: 10.3389/fonc.2024.1463173 (PMC11540557; doi:10.3389/fonc.2024.1463173)
Supplement: Supplementary file 1 [file DataSheet1.docx]

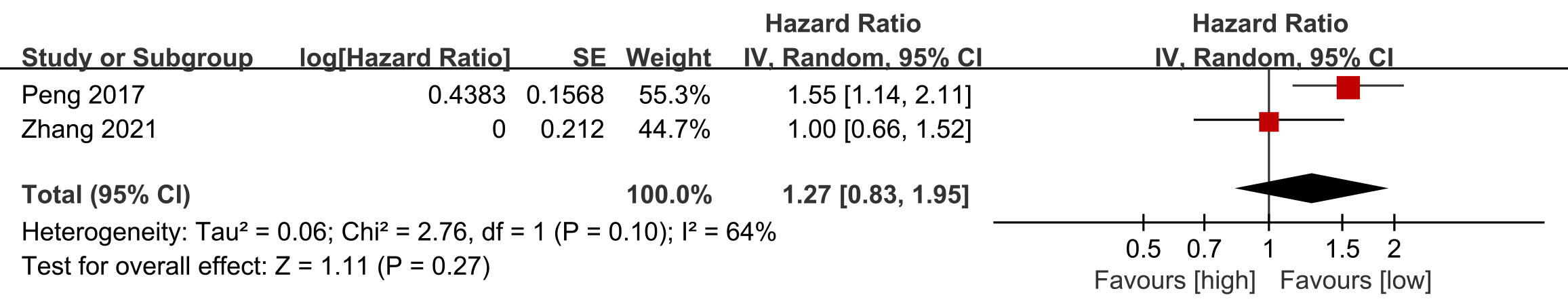


Figure S1: Forest plots of PFS.


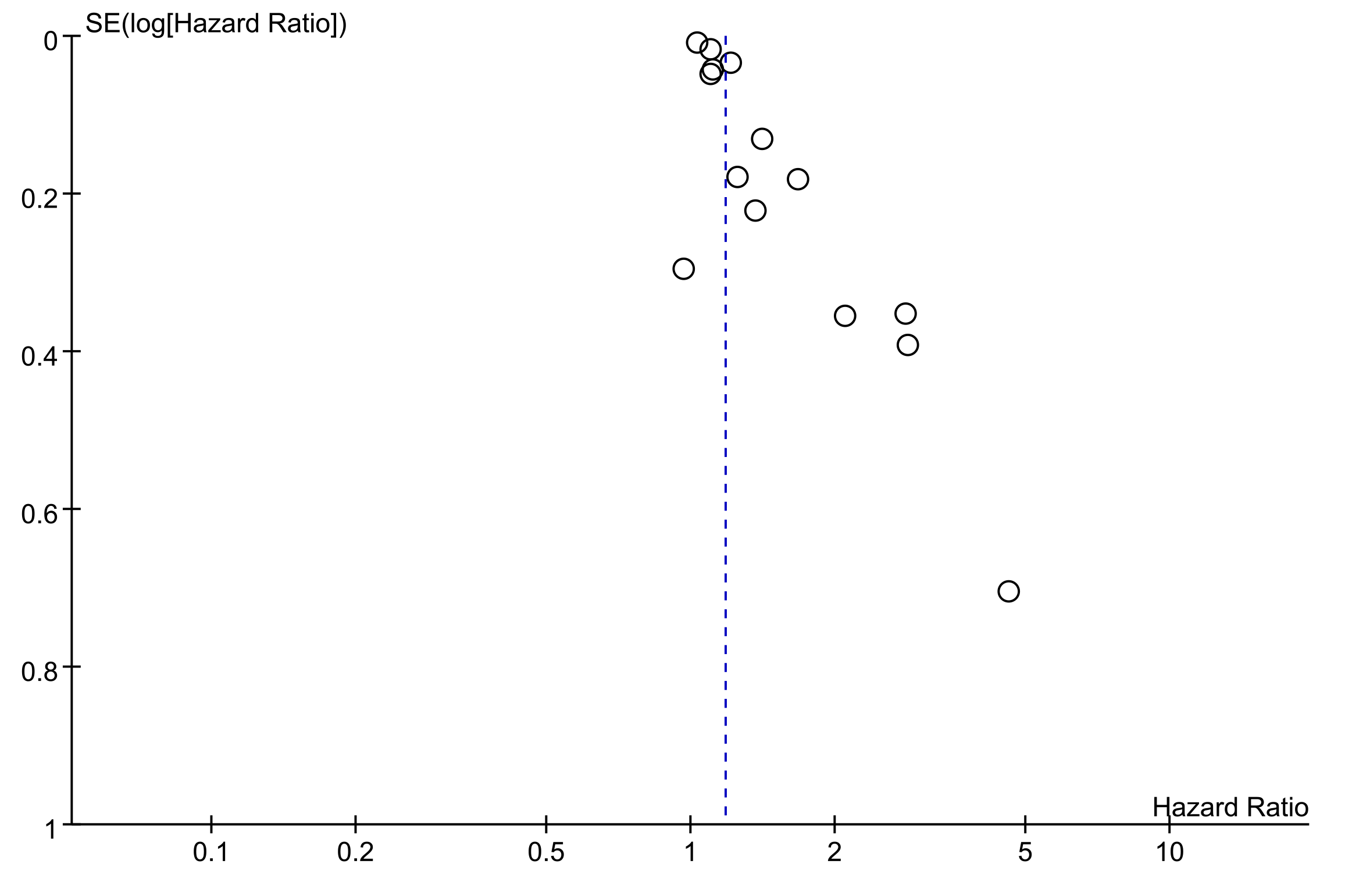


Figure S2: Funnel plots of OS.


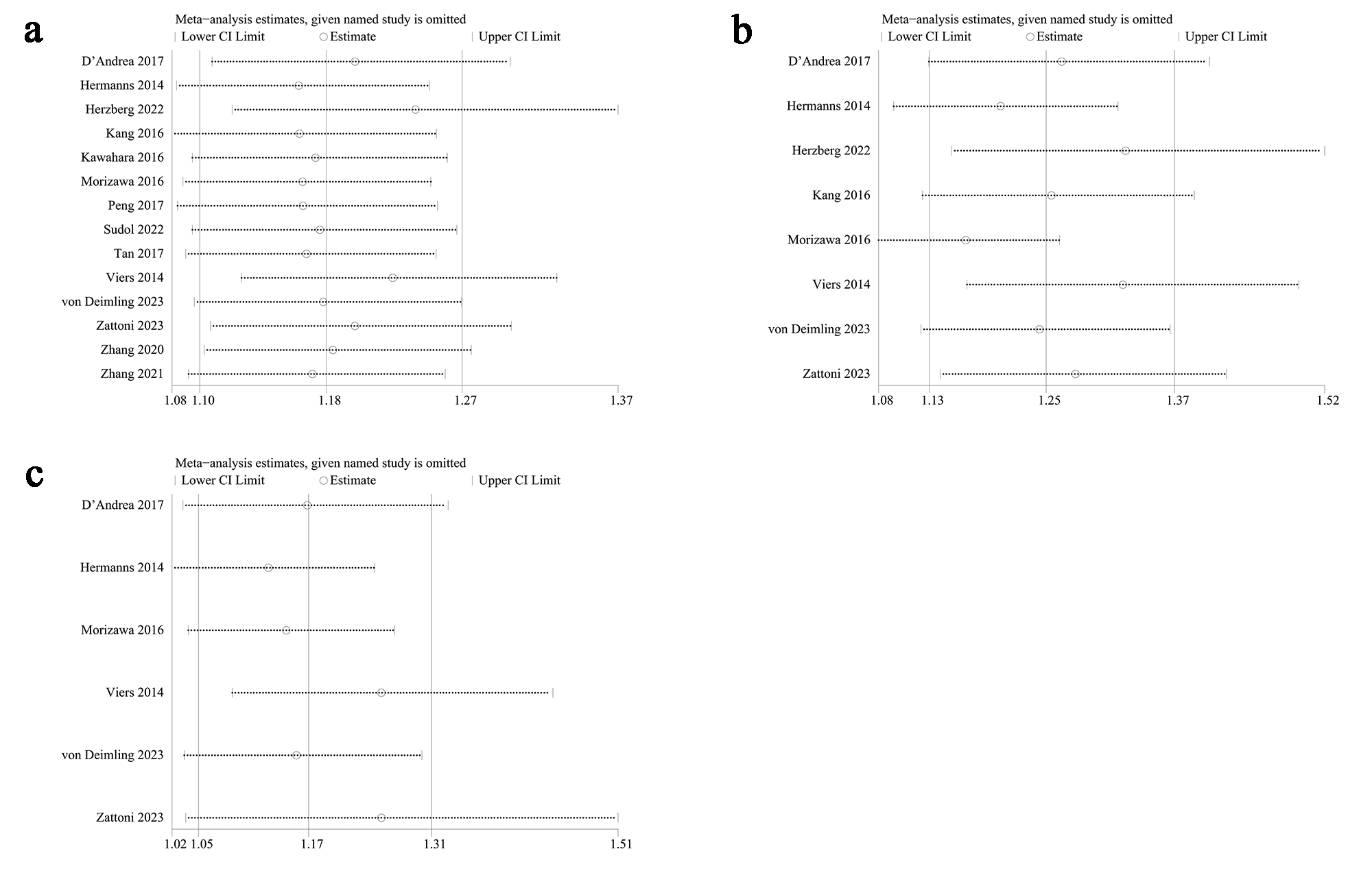


Figure S3: Sensitivity analysis of OS (A), CSS (B) and RFS (C).
